# Fast Thermal Resistance Distribution Analysis in High-Power VCSEL Array Module

**DOI:** 10.3390/ma18225210

**Published:** 2025-11-17

**Authors:** Dezhen Li, Tian Lan, Zhiyong Wang, Zhengyu Ye

**Affiliations:** 1 Institute of Advanced Technology on Semiconductor Optics & Electronics, School of Physics and Optoelectronic Engineering, Beijing University of Technology, Beijing 100124, China; lidezhen@emails.bjut.edu.cn (D.L.); zywang@bjut.edu.cn (Z.W.); 2 Key Laboratory of Trans-Scale Laser Manufacturing Technology (Beijing University of Technology), Ministry of Education, Beijing 100124, China

**Keywords:** VCSEL array module, thermal resistance, electrical transient measurement, structure function, spectroscopy method

## Abstract

**Highlights:**

**What are the main findings?**

- Thermal resistance analysis can be used for a semiconductor laser module.
- Thermal resistance tests occur within extremely short times (25 s).
- Segmented thermal resistance distributions for the module are obtained.

**What are the implications of the main findings?**

- This study reveals a trustworthy and rapid thermal resistance analysis method for the VCSEL array module.
- This electrical transient measurement can identify the thermal resistance link of the module without any damage.
- Thermal resistances from R_submount_ and R_solder2_, which account for 54% of the total thermal resistance, have great potential for improvement.

**Abstract:**

Vertical-cavity surface-emitting lasers (VCSELs) have generated extensive enthusiasm in scientific research on and applications of lasers. However, thermal resistance has seriously limited the performance of such devices for a long time, especially in high-power single-chip large-area VCSEL array modules. In this study, in order to determine the packaging thermal resistance bottleneck of the high-power VCSEL array laser module and achieve better performance, the thermal characteristics of an 808 nm VCSEL module were analyzed quickly with electrical transient measurements without any damage, which consisted of a 6 mm × 6 mm, 85 W, AlGaAs/GaAs VCSEL array chip encapsulated on a submount and a water-cooled heat sink. The quantitative components of the device’s thermal resistance were clearly segmented and rapidly obtained within merely 25 s using the structure function algorithm. The packaging thermal resistances together accounted for an astonishing 70% of the total thermal resistance when the loading current was 8 A. Among them, R_submount_ and R_solder2_ were the main focus areas, which accounted for 54% of the total thermal resistance. We also applied the spectroscopy method to calculate the total thermal resistance of the module on a large scale from another perspective for the comparative verification of the electrical transient method. The values obtained by the two methods were relatively close. More importantly, this research will have a positive impact and an indicative effect on reducing the main thermal resistances of the VCSEL array module.

## 1. Introduction

**Figure 1:**
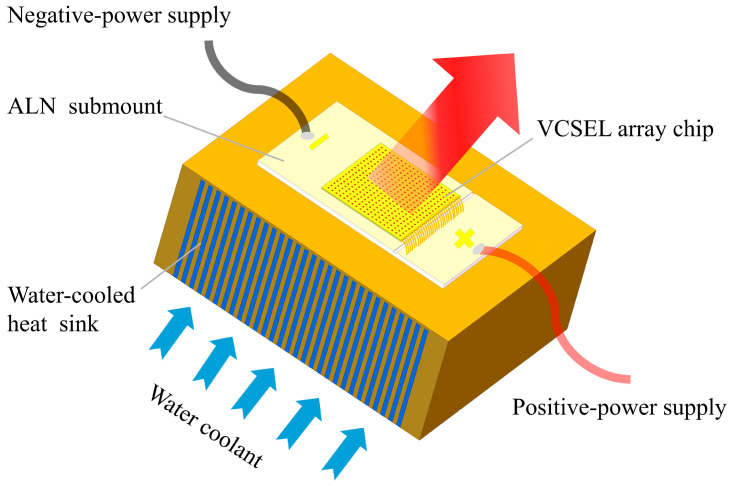
Operating principle of high-power VCSEL array module with water cooling.

**Figure 2:**
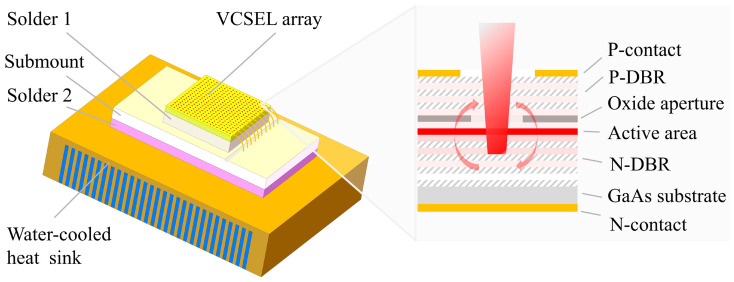
Operating principle of VCSEL cell in module.

In recent years, the vertical-cavity surface-emitting laser (VCSEL) has emerged as a highly promising laser that is extensively applied in industry, consumer electronics, medical treatment, and military applications, among others [1,2,3]. To meet the demand for high-power lasers, a single VCSEL array chip with tens of thousands of luminous points within only a few millimeters has been developed, which has a laser power from tens to hundreds of watts [1]. The operating principle of the high-power VCSEL array module is depicted in Figure 1. The operating principle of the VCSEL cell in the module is depicted in Figure 2. However, such a chip design also creates a serious heat accumulation problem in the VCSEL array [4]. This situation severely impairs the performance of the VCSEL array, resulting from increased threshold current, reduced electro-optical conversion efficiency, and impaired working life [5].

Thermal resistance analysis is extremely instructive, which is one of the most useful indicators for semiconductor lasers [6,7]. Thermal management for the VCSEL array is especially important. However, the exact thermal resistance distribution of the VCSEL array module with cooling is still unclear [5]. The results of finite element simulation are overly idealized and neglect the thermal resistances within the interface transition areas, which leads to the simulation-predicted thermal resistance being usually lower than the actual thermal resistance. In addition, the VCSEL array chip with extremely complex micro/nano architectures is difficult to accurately replicate. Furthermore, simulation consumes a great deal of time, i.e., several days [7,8,9]. The electrical measurement technique is a popular method for obtaining and analyzing the thermal resistance of various power devices such as an insulated gate bipolar transistor (IGBT) [10,11,12,13]. More importantly, this method is convenient and accurate. It does not cause any damage to devices and can be used to obtain the exact distribution of various thermal resistances of the devices under test. However, the electrical measurement technique can assess the thermal resistance compositions of only the water-cooling part of the module and not the whole module due to its limited actual heat capacity. The spectroscopy method can serve as an important supplementary method to obtain the overall device thermal resistance of the laser module. As stated above, an accurate and quantitative thermal resistance analysis of the high-power VCSEL array module plays a vital role and has a positive effect on improving its performance.

In this study, we have combined the electrical transient method and the spectroscopy method to quickly and accurately ascertain the thermal resistance distribution of the VCSEL array module. Both of these methods do not cause any damage to the module. The electrical transient method is very practical and is based on the linear relationship between the forward voltage and the semiconductor chip’s junction temperature. Using this method, we can ascertain the thermal resistance distribution corresponding to the main components of the module. We have carefully analyzed the thermal resistance distribution in the VCSEL array module at different loading currents using the structure function method. The spectroscopy method is based on the relationship between bandgap and junction temperature based on semiconductor laser chips. This method can accurately ascertain the total thermal resistance of the semiconductor laser module across the entire current range. Thus, we applied this method to determine the total thermal resistance of the module and compared it with the electrical transient method. The combination of the two methods is mutually beneficial to dialectically analyze the thermal resistance compositions of the VCSEL array module.

## 2. Materials and Methods

### 2.1. Materials’ Introduction

**Figure 3:**
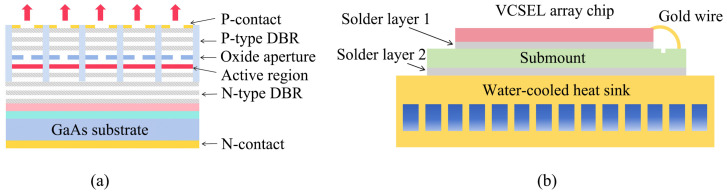
(**a**) Schematic of VCSEL array chip. (**b**) Schematic of packaged VCSEL array module.

The 808 nm, 6 mm × 6 mm, AlGaAs/GaAs top-emitting VCSEL array chip’s main schematic is shown in Figure 3a. The spacing between the VCSEL arrays is about 25 μm. The VCSEL array chip is mainly made of a P-contact electrode layer, a P-type distributed Bragg reflector (DBR), an oxide aperture, an active region, an N-type DBR, a GaAs substrate, an N-contact electrode layer, etc. The schematic of the VCSEL array module with a remote cooling packaging style is shown in Figure 3b.

The module consists of a single VCSEL array chip, a special solder layer 1, an aluminum nitride heat sink submount, a special solder layer 2, and a water-cooled heat sink. The packaging form is usually of remote cooling-type, which has been widely used for high-power VCSEL array modules since a long time. In this study, the chip with thousands of luminous points is thinned to no more than 100 μm and then metallized. Nano silver sintering is used between the VCSEL array chip and the submount to ensure no void is present inside the whole solder layer, which is extremely important because a void causes excessive thermal resistance, resulting in the local burning of the VCSEL array. The thickness of the solder layer 1 is less than 5 μm, which is achieved at 20 Mpa, 200 °C, and 90 min. At the same time, the solder layer’s thermal conductivity can reach up to 200 W/m·K, which is a satisfactory thermal interface. The submount is made of aluminum nitride material that has a thermal expansion coefficient similar to gallium arsenide and relieves packaging stress. The double-side welding method using indium is utilized to connect the submount and the water-cooled heat sink. On the one hand, this method is used to reduce voids in the solder layer 2. On the other hand, it also alleviates packaging stress due to the presence of indium, which is soft and usually as a suitable solder for a laser module. The thickness of solder 2 is about 10 μm. The water-cooled heat sink is made of purple copper, which is processed by wire electrical discharge machining to form a multi-channel array. Thus, we can enhance the ability of convective heat transfer with water or other coolants to dissipate the heat of the module. The channel width of the water-cooled heat sink is about 300 μm, and the channel width-to-wall thickness ratio is about 1:1 in the water-cooled heat sink. The main elements and parameters of the VCSEL array module are described in Table 1.

**Figure 4:**
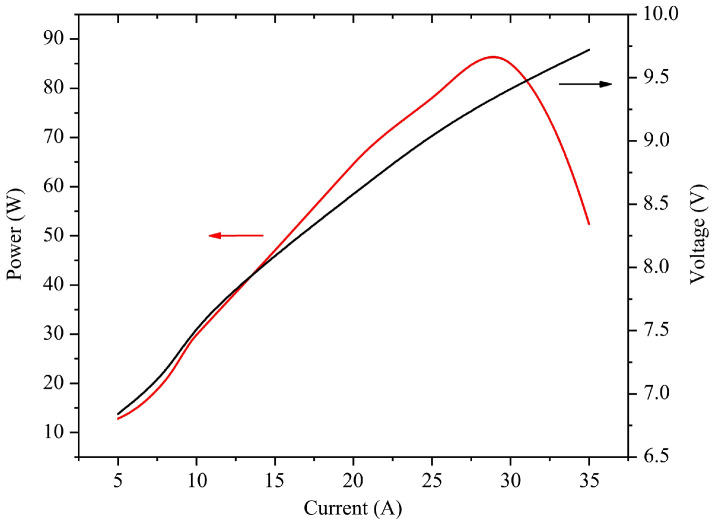
PIV curve of the VCSEL array module.

We evaluated the performance of the packaged module. The threshold current of the chip was about 5 A. The power/current/voltage (PIV) chart is shown in Figure 4; the conditions during the power/current/voltage assessment were as follows: the module was in continuous wave operation on a water-cooled heat sink, the water temperature was set at around 20 °C, and the water flow rate was set at 2 L/min to maintain the flow state in turbulence.

**Figure 5:**
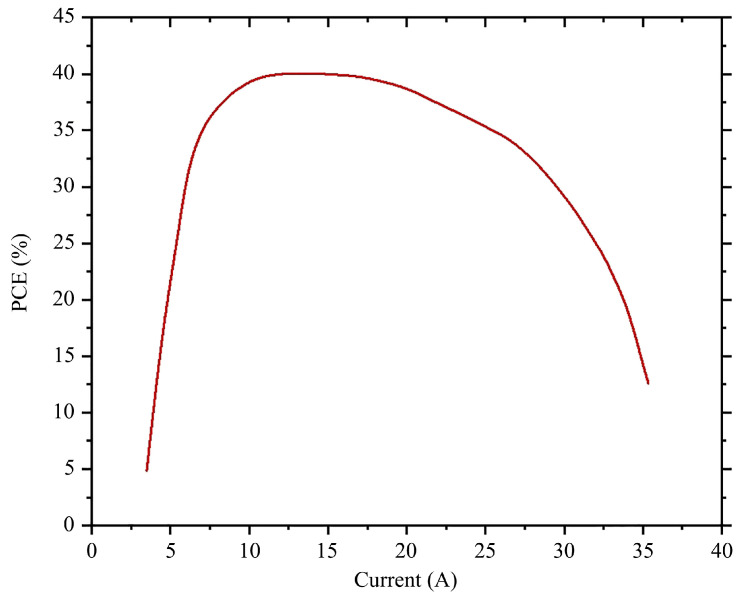
Power conversion efficiency (PCE) curve of the VCSEL array module.

A laser power tester was used for laser power detection, which is based on the principle of thermo-electric conversion. When the current was set at about 27 A, the laser power of the module reached the peak power of about 85 W. Then, the output laser power of the module began to decrease due to the insufficient heat dissipation of the remote cooling module. To more intuitively reflect the thermal resistance effect of the module, the power conversion efficiency (PCE) curve is shown in Figure 5.

The maximum electro-optic conversion efficiency approached 40% of the module and occurs at a loading current of 9 A~15 A. Thereafter, the efficiency started to rapidly decline as the loading current gradually increased. As mentioned above, the thermal resistance effect severely limits the electro-optic conversion efficiency of the module. Therefore, the detailed thermal resistance analysis of the VCSEL array module is extremely meaningful.

Thermal resistance is a key indicator of thermal performance in semiconductor lasers. The JEDEC standard No. 51-1 defines thermal resistance as follows [14]:

(1) $$R_{th}=\frac{∆T}{P_{t}}=\frac{T_{j}-T_{a}}{P_{t}}$$

In Equation (1), ***P_t_*** refers to the heat dissipation power, ***T_j_*** refers to the junction temperature of the chip, and ***T_a_*** refers to the ambient temperature. When the VCSEL array is loaded with the input current, heat generates inside the chip and packaging structure, which exhibits joule heating, scattering absorption, reabsorbed component of spontaneous emission, carrier leakage, band alignment, etc. Once the loading current exceeds the threshold current ***I_th_***, the VCSEL array chip emits a laser beam.

### 2.2. Structure Function Method

**Figure 6:**
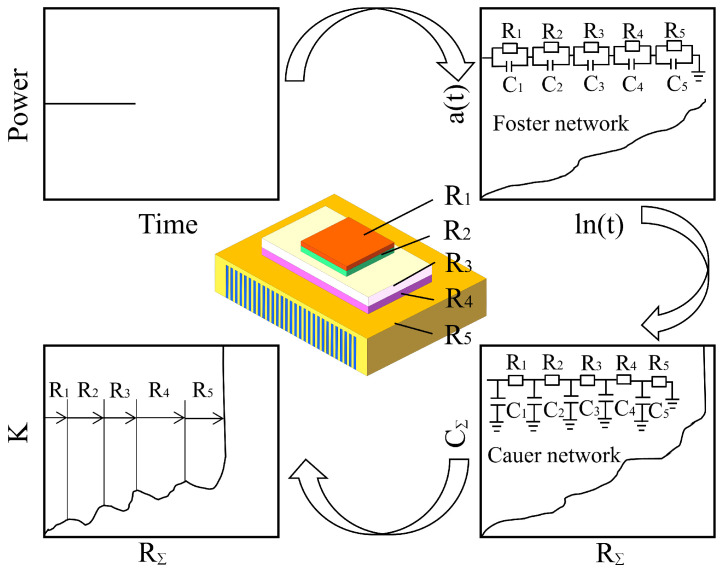
Structure function flow chart.

The structure function method for measuring thermal resistance refers to utilizing the linear relationship between the voltage and the temperature of the semiconductor junction. The structure function algorithm flow chart can be divided into the following steps: Firstly, the transient temperature rise response curve was obtained by detecting the device under test, which was based on temperature-sensitive parameters and their real-time variation curves. Secondly, we obtained a time-constant spectrum through deconvolution calculations. Thirdly, we obtained the Foster thermal resistance and heat capacity network model through discretization. Fourthly, we transformed it into a more practical and meaningful Cauer module. Fifthly, we determined the heat capacity and thermal resistance to obtain the integral structure function curve. Sixthly, we obtained the differential structure function curve through differentiation. The above-described flow chart is depicted in Figure 6.

During the heating or cooling process of the semiconductor laser, the transient temperature curve can be obtained by rapidly measuring the junction voltage of the device. With the thermal RC network analysis theory, the transient temperature response curve is analyzed for a thermal RC network, and then transformed into a differential structure function curve using mathematical processing.

Thus, a detailed analysis of the thermal resistance of each layer in the device packaging can be adequately performed [15]. Semiconductor devices are generally packaged into multi-layer structures composed of different materials [16]. Within the thermal conduction path of a device, different materials exhibit varying thermal resistances and thermal capacities, leading to distinct temperature responses. Therefore, semiconductor devices and their packaging structures can be regarded as one-dimensional thermal networks. At *t* = 0, we apply heat power to the device, then the junction voltage of the device is measured to obtain the curve of temperature variation with time during the temperature rise, which is called the transient response curve. This curve is equivalent to the response of a Foster RC network model with n units. This response can be expressed as a linear combination of exponential terms with n different time constants:

(2) $$a\left(t\right)=\sum_{i=1}^{n}R_{i}\left[1-exp(-t/\tau_{i})\right]$$

In Formula (2), $a\left(t\right)$ represents the thermal response curve, $\tau_{i}$ = $R_{i}$ ***×*** $C_{i}$ denotes the time constant of No_i_ RC, and ***R_i_*** refers to the amplitude coefficient of the exponential term in the time-constant spectrum. During the measurement process, the time range is large, and for the convenience of mathematical calculations, the natural logarithm of time is usually taken, i.e., ***z*** *=* ***lnt***. In the thermal conduction system of semiconductor systems, discrete thermal conduction networks have the characteristic of continuous time-constant spectra, and a time-constant spectrum is defined as follows:

(3) $$R(z)=\underset{\delta_{z}\to 0}{\lim}\frac{the\;amplitude\;of\;the\;thermal\;time\;constant\;between\;z\;and\;z+\delta_{z}}{\delta_{z}}$$

Therefore, ***a*(*t*)** can be transformed to the following form:

(4) $$a(z)\;=\;\int_{-\infty }^{+\infty }R(\zeta )\left\{1-exp\left[-exp(z-\zeta )\right]\right\}d\zeta$$

In Formula (4), ζ = ***ln***$\tau_{i}.$ For the spectrum of unknown time constants R(ζ), Formula (4) is a devolution-type differential equation. Taking the derivative of both sides with respect to ***z***, we obtain the following equation:

(5) $$\frac{d}{dz}a(z)=R(z)⊗W(z)$$

In Formula (5), ***W*(*z*)** = ***exp***$\left[z-exp(z)\right]$, and ⊗ represents a convolution operation. Therefore, after measuring the thermal response curve, it is transformed into an exponential time variable form. Take its derivative and by performing a deconvolution operation based on Equation (5), we can obtain the time-constant spectrum of the one-dimensional thermal structure R(z). Thus, the thermal resistance ***R_i_*** and thermal capacity ***C_i_*** in the Foster network can be extracted. In fact, the point-to-point heat capacity in the Foster network has no practical physical significance. Therefore, it is necessary to transform the Foster network into the Cauer network with a point-to-ground thermal capacity model. The N-piece RC units obtained from the Cauer network can be used to approximately describe the heat transfer structures of the device. The thermal response curve of the junction temperature is determined by the structure function of the device. In addition, the structure function is defined as the ratio of heat capacity per unit length to the thermal resistance per unit length, as follows:

(6) $$k(x)=\frac{c(x)}{r(x)}$$

In the above formula, ***x*** denotes the linearity of the one-dimensional thermal conduction path. Along the heat transfer path, we sum up the thermal resistance and heat capacity of each RC unit to obtain $R_{\Sigma }$**(*x*)** and $C_{\Sigma }$**(*x*)**. By comparing with them, the distribution of the structure function along the thermal conduction path ***x*** can be obtained. In fact, the commonly used structure in thermal resistance test instruments is the differential structure function, which is the function value obtained by differentiating $C_{\Sigma }$**(*x*)** with respect to $R_{\Sigma }$**(*x*)**:

(7) $$K[R_{\Sigma }(x)]=\frac{dC_{\Sigma }(x)}{dR_{\Sigma }(x)}$$

We consider a thin material with a cross-sectional area of A and a thickness of dx. Its heat capacity can be represented as ***d***$C_{\Sigma }$ = ***c_v_Adx***, where **c_v_** represents the volumetric heat capacity. The thermal resistance can be represented as ***d***$R_{\Sigma }$ *=* ***dx/eA***, where ***e*** denotes the thermal conductivity of the material. Therefore, the differential structure function of Equation (7) can be expressed as follows:

(8) $$K[R_{\Sigma }(x)]=\frac{dC_{\Sigma }(x)}{dR_{\Sigma }(x)}=\frac{C_{v}Adx}{dx/eA}=\;c_{v}eA^{2}$$

For a certain structure along the heat flow path, both ***c_v_*** and ***e*** are constants, while the heat flow cross-sectional area A gradually increases due to thermal diffusion effects. There are local peaks or troughs on the curve of the differential structure function. This is caused by the sudden changes in ***c_v_*** and ***e***, indicating that the heat flow has reached a new material. Therefore, by analyzing the curve of the differential structure function, we can identify and analyze the internal layer structures of the devices and their thermal conductivity characteristics [16].

The temperature rise and the thermal resistance in the VCSEL array are measured with the electrical transient measurement technique. This is based on the linear relationship between the forward voltage and the junction temperature variation, which is defined as follows [17]:

(9) $$∆T=\frac{{∆V}_{pn}}{k}$$

**Figure 7:**
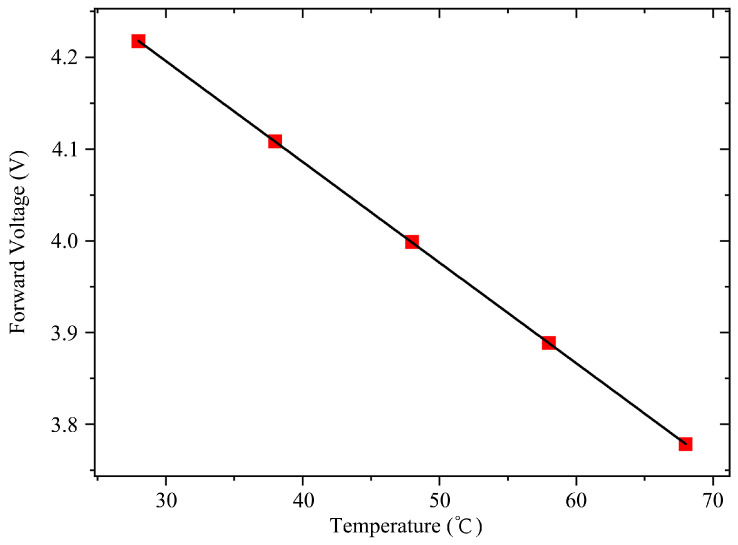
Forward voltage as function of temperature.

In the above equation, **△*V_pn_*** represents the forward voltage difference in the VCSEL array before and during operation, and k represents a temperature coefficient. We measured the forward voltages at a steady diminutive current of 1 mA under various temperatures from 28 °C to 68 °C, at a rate of 10 °C per step. The current was loaded with the Keithley 2400 source meter, which can maintain the stability of the testing data. The ambient temperature was regulated and maintained using a constant-temperature bath, and the temperature tolerance was controlled within ± 0.05 °C. Then, we obtained a clear linear proportionality constant, which is about −11 mV/°C for the tiny current of 1 mA, as shown in Figure 7.

**Figure 8:**
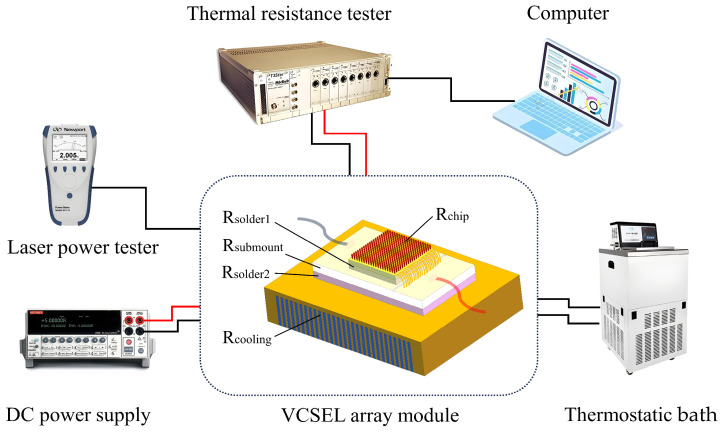
Thermal resistance-testing system.

The thermal resistance of the module was evaluated using a thermal resistance tester at working currents from 2 A to 8 A with IT6722A DC power supply at a rate of 2 A per step, which is around the threshold current of 5 A of the top-emitting VCSEL array chip. The module was set at a stabilized temperature of about 25 °C by using a thermostatic bath during the thermal transient measurement, as shown in Figure 8. 

***P_o_*** was determined with a handheld laser power energy meter, when the VCSEL array module was loaded with the operating current during the thermal resistance-testing process. In addition, the thermal power was calculated using the following equation:

(10) $$P_{t}=I\times V-\;P_{o}$$

**Figure 9:**
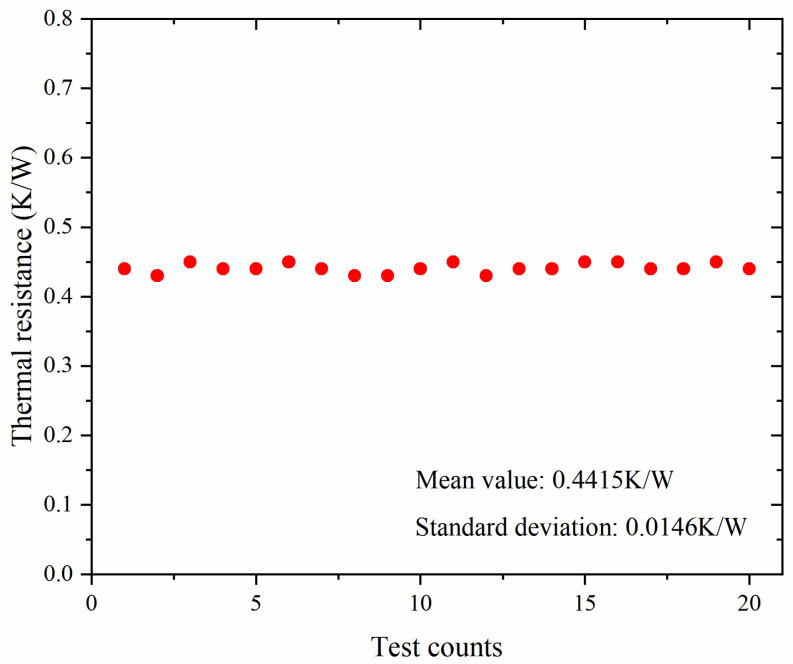
Total thermal resistance and deviation tested twenty times.

In the above equation, ***P_t_*** represents the thermal power, ***I*** represents the operating current, ***V*** represents the operating voltage, and ***P_o_*** represents the laser power. The VCSEL array was operated at a set current within only 15 s, which is enough to keep the device in a steady state. Then, it was quickly switched from a working state to a measurement state to check the forward voltage under a sensor current of only 1 mA. In addition, due to the fast cooling of the water-cooled module, the cooling time for testing was completed within merely 10 s. The switching process was quick enough, i.e., less than 1 μs. We can obtain the thermal resistance curve and the transient cooling curve using Equations (1) and (9). The transient cooling curve can be switched to a transient temperature rise curve using a mathematical inversion [17,18,19,20]. To obtain more detailed information on the heat conduction process of the module, we analyzed its thermal resistance composition using the transient temperature rise curve with the structure function algorithm. We measured the thermal resistance data of the module up to 20 times at 8 A, and the total thermal resistance data remained stable. The total thermal resistance and deviation tested twenty times are depicted in Figure 9.

### 2.3. Spectroscopy Method

The structure function method has considerable advantages in detecting the composition of the module’s thermal resistance. However, this method cannot accurately detect the thermal resistance of the overall water-cooling laser module. In this regard, the spectroscopy method is more suitable. GaAs semiconductor lasers are made of direct bandgap materials: the junction temperature changes linearly with laser wavelength, which is defined as follows [21]:

(11) $$∆T=\frac{∆\lambda }{\lambda (T)}$$

**Figure 10:**
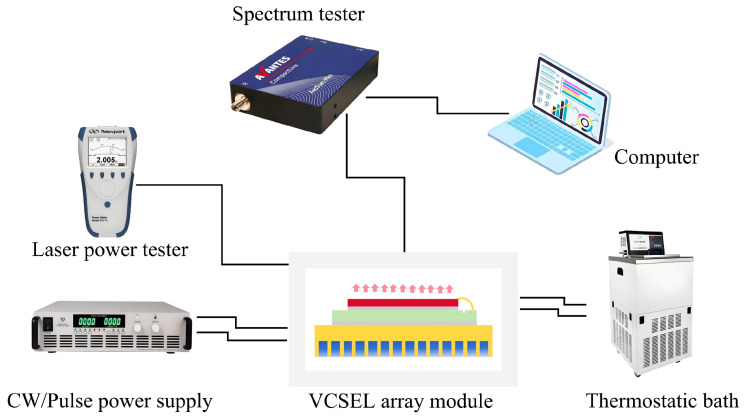
Spectroscopy method testing system.

In the above equation, **∆*T*** denotes the junction temperature variation in the laser chip, **∆*λ*** denotes the wavelength variation, and ***λ*(*T*)** denotes the temperature drift coefficient. Firstly, we obtained the temperature drift coefficient by observing the variation in pulse laser wavelength at 5 A, 100 μs, 100 Hz, and a stable junction temperature by gradually changing the environmental temperature. The ambient temperature was adjusted and controlled by a thermostatic bath instrument, as shown in Figure 10.

**Figure 11:**
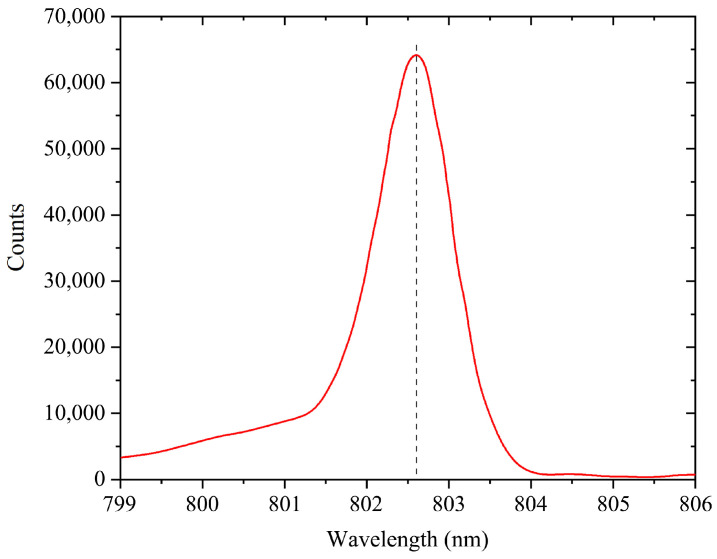
Spectral distribution at 25 °C and low-duty pulse working state.

**Figure 12:**
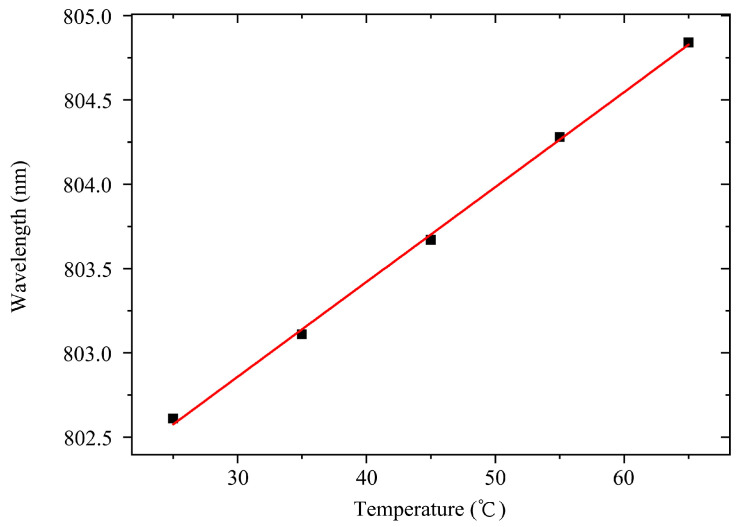
Wavelength as function of temperature.

We utilized the Avantes spectrometer to monitor the laser wavelength. Due to the high power of the laser, we used the laser reflection method with a metal plate above to reduce the overly high laser spectral intensity required for detection to avoid instrument damage. The spectral distribution test results at 25 °C are shown in Figure 11. The central wavelength at the low duty cycle is approximately 802.60 nm, which is commonly known as the cold temperature wavelength. We transformed the test data using the software OriginPro to obtain the current spectral distribution, as shown in Figure 11. As depicted in Figure 12, the temperature drift coefficient is about 0.056 nm/K.

Then, we employed the wavelength drift method to monitor the laser wavelength variation with various operating currents, thereby inferring the junction temperature rise with thermal power, and subsequently calculating the total thermal resistance of the device. To obtain convenient detection and calculation for the thermal resistance of the laser module, Equation (1) can be transformed into the following form [22]:

(12) $$R_{th}=\frac{△T}{△P_{t}}=\frac{△T}{△\lambda }\frac{△\lambda }{△P_{t}}$$

In the above equation, ***R_th_*** denotes the thermal resistance, and ***P_t_*** denotes the thermal power. We assessed the voltage, the laser power, and the wavelength of the VCSEL array module at different loading currents, i.e., 5 A~35 A, in a continuous working condition. These test parameter data are shown in Table 2.

## 3. Results and Discussion

### 3.1. Thermal Resistance Analysis Using Structure Function Method

**Figure 13:**
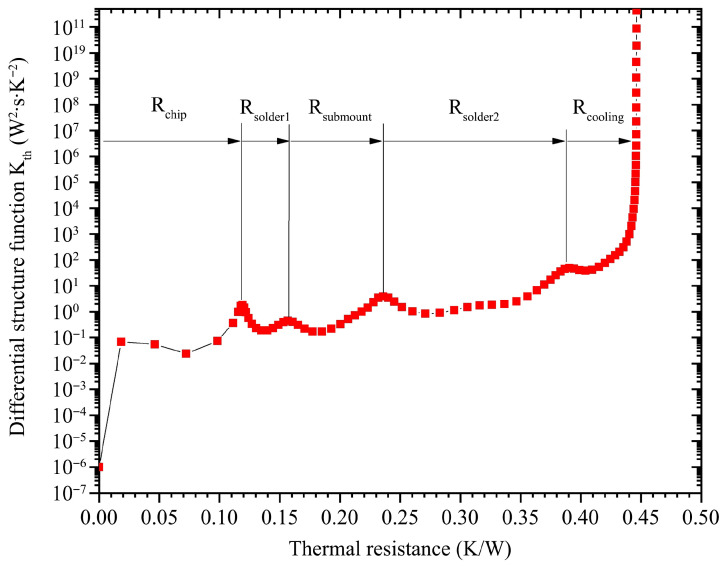
Differential structure function at loading current of 8 A.

According to the structure function algorithm flow chart mentioned in Section 2.2, we obtained the differential structure function curves through thermal resistance tester and specialized software calculations. Figure 13 shows the differential structure function of the VCSEL array module at a loading current of 8 A, and the total thermal resistance of the module is nearly 0.44 K/W. There are five peaks that correspond to the thermal resistance of the VCSEL array chip, R_chip_; the solder layer 1, R_solder1_; the aluminum nitride submount, R_submount_; the solder layer 2, R_solder2_; and the resistance of the cooling module, R_cooling_, respectively.

**Figure 14:**
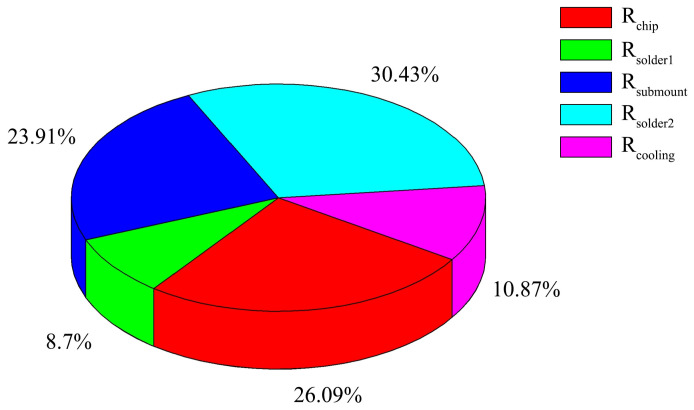
Thermal resistance distribution of module at loading current of 8 A.

Meanwhile, the total thermal resistance obtained from Table 1 is about 0.40 K/W at 8 A, which is close to that obtained from Figure 13. Using the differential structure function, we found that every part’s segmented thermal resistance corresponds to the device composition, and the thermal resistances are dominated by the packaging design. The encapsulation thermal resistances together account for over 70% of the total thermal resistance of the module. The thermal resistance distribution of the module at a loading current of 8 A based on Figure 13 is shown in Figure 14.

**Figure 15:**
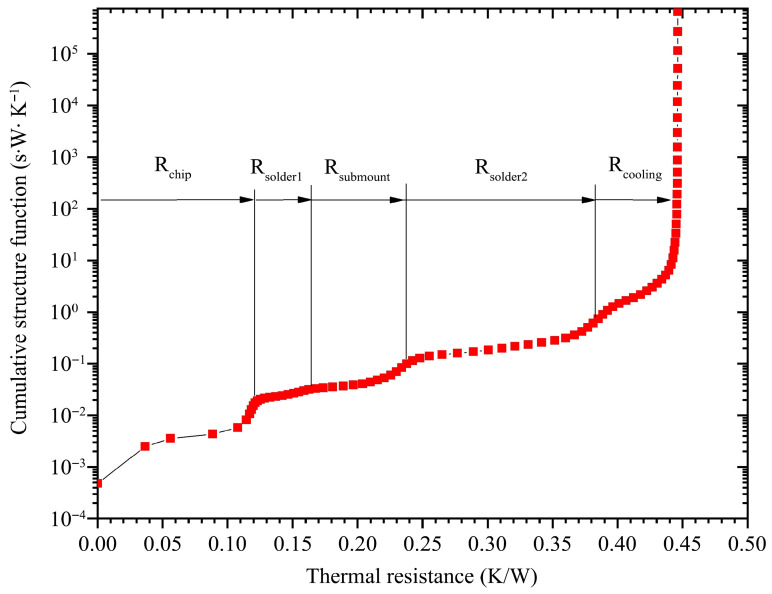
Cumulative structure function at loading current of 8 A.

Similarly, Figure 15 shows the cumulative structure function analysis of the VCSEL array module at the same loading current of 8 A. This form also plainly presents the thermal resistance accumulation of the module from another perspective. The above analysis helps us to carry out the task of reducing thermal resistance with a clear objective.

Methods such as wafer bonding may be an effective approach for reducing interfacial thermal resistance between the chip and the submount. Furthermore, the direct formation of an embedded microchannel inside the submount for the module effectively reduces the thermal resistances from R_submount_, R_solder2_, and R_cooling_. With the above improvements and efforts, even with a high thermal power density as high as hundreds of kilowatts per square centimeter, the junction temperature of high-power laser chips will be ultimately reduced due to our continuous efforts.

### 3.2. Thermal Resistance Analysis Using Spectroscopy Method

**Figure 16:**
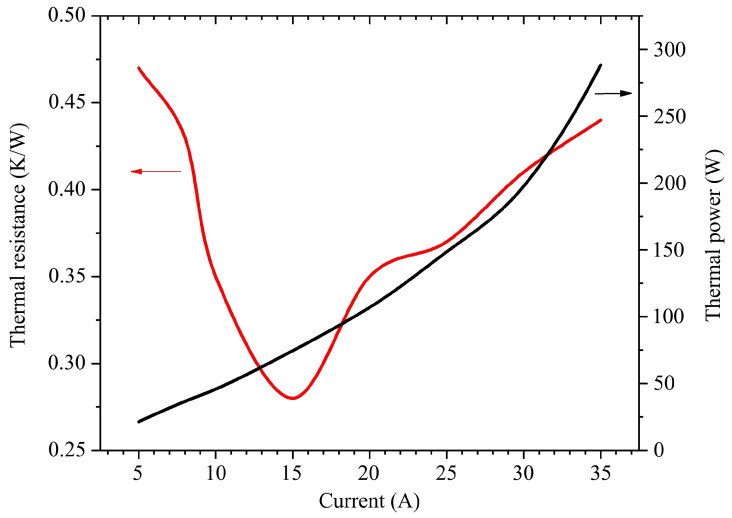
Thermal resistances and thermal powers as function of various loading currents.

The data from the differential structure function do not allow us to obtain accurate thermal resistance values for the water-cooled heat sink or the total thermal resistance of the module. Furthermore, in this study, the upper current limit of the thermal resistance tester using the structure function algorithm is 10 A; the tester cannot cover the entire current range from 0 A to 35 A. Therefore, we attempted to analyze the overall thermal resistance of the VCSEL array module using the spectroscopy method. Based on the data from Table 1, we calculated the thermal resistance with Equations (10) and (12). Furthermore, we obtained the correlations among the thermal resistance, thermal power, and loading current, as depicted in Figure 16.

As the loading current increases, the thermal power of the laser module rapidly increases. The thermal resistance of the laser module is generally between 0.3 K/W and 0.5 K/W and not constant like the chip’s work status. When a current of 15 A is applied, the lowest thermal resistance of the device is approximately 0.27 K/W. Correspondingly, as depicted in Figure 3, the power conversion efficiency reached its peak nearly in the same interval. This may be due to the following two reasons: Firstly, the chip works best in this range. Secondly, the cooling capacity of the packaging structure can effectively control the temperature of the module within this thermal power range. Afterwards, as the thermal power increases, the heat dissipation level of the cooling module becomes even capable of meeting the heat dissipation requirement. The chip junction temperature begins to continuously rise. Then, the module begins to show a thermal rollover. This also highlights the importance of changing the packaging structure and improving the heat dissipation level for the VCSEL array module to reduce its thermal resistance effect.

## 4. Conclusions

The thermal resistances of the 808 nm high-power AlGaAs/GaAs VCSEL array module were investigated using the electrical transient measurement and spectroscopy methods. We systematically studied the thermal resistance distribution and bottleneck of the traditional water-cooled high-power VCSEL module, providing direction for improving the heat dissipation of the high-power VCSEL module. The quantitative data pertaining to the thermal resistance values of the module were measured and quickly obtained within less than 0.5 min. The structure function algorithm was applied to ascertain the quantitative data of the thermal resistance compositions. The study results reveal that the total thermal resistance mainly results from the solders and the submount, which ultimately causes a significant increase in the junction temperature of the VCSEL array chip. The two methods comprehensively explained the influence of thermal resistance on the VCSEL array module from two different perspectives. The total thermal resistance of the module is approximately 0.44 K/W and not constant as the chip’s real-time mode. When the loading current is 8 A, the packaging thermal resistance accounts for as much as 70% of the total thermal resistance of the module. R_submount_ and R_solder2_ are the main components that account for about 54% of the total thermal resistance.

The above quantitative analyses provide accurate results and constructive suggestions for future research on reducing the thermal resistance of the high-power VCSEL module. Thus, there is a lot of room for optimally reducing the thermal resistance of the VCSEL array module. The thermal resistances R_submount_ and R_solder2_ are the foci, which need to be significantly reduced. The solder may be replaced by wafer bonding to significantly reduce R_solder1_. Integrating manifold microchannel cooling into the submount may significantly reduce the thermal resistance of the VCSEL array module.

## Figures and Tables

**Table 1:** Main elements of VCSEL array module.

	Elements	VCSEL Array	Solder 1	Submount	Solder 2
Parameters					
Main material		GaAs	Ag	ALN	Indium
Thickness (μm)		100	5	350	10
Area (mm^2^)		36	36	112	112
Thermal conductivity (W/m·K)		56	200	180	82

**Table 2:** Test parameter data of VCSEL array module in continuous working condition.

Current (A)	Voltage (V)	Laser Power (W)	Wavelength (nm)	Thermal Power (W)
5	6.84	12.8	803.16	21.4
8	7.14	20.5	803.41	36.6
10	7.56	29.8	803.50	45.8
15	8.10	47.0	803.78	74.5
20	8.58	64.6	804.67	107.0
25	9.06	78.0	805.73	148.5
30	9.42	85.0	807.13	197.6
35	9.72	52.4	809.58	287.8

## Data Availability

The original contributions presented in this study are included in the article. Further inquiries can be directed to the corresponding authors.
